# Breaking Specialty Silos: Improving Global Child Health Through Essential Surgical Care

**DOI:** 10.9745/GHSP-D-20-00009

**Published:** 2020-06-30

**Authors:** Isaac Wasserman, Alexander W. Peters, Lina Roa, Farhana Amanullah, Lubna Samad

**Affiliations:** a Icahn School of Medicine at Mount Sinai, New York, NY, USA.; b Program in Global Surgery and Social Change, Department of Global Health and Social Medicine, Harvard Medical School, Boston, MA, USA.; c Department of Plastic and Oral Surgery, Boston Children’s Hospital, Boston, MA, USA.; d Department of Surgery, Weill Cornell Medical College, New York, NY, USA.; e Department of Obstetrics & Gynecology, University of Alberta, Edmonton, Canada.; f The Indus Hospital, Karachi, Pakistan.; g Center for Essential Surgical and Acute Care, Indus Health Network, Karachi, Pakistan.

## Abstract

Children’s health care providers and children’s surgery providers can partner to improve children’s health by developing the surgical workforce, focusing on “best buy” surgeries, integrating children’s surgery into national plans, streamlining data collection and research, and leveraging financing.

## Key Messages

- Despite the large role that children’s surgery plays in reducing morbidity and mortality, global child health initiatives have historically focused on nonsurgical diseases.
- Children’s health care providers and children’s surgery providers can collaborate to improve children’s health through shared values.
- Long-term investments in surgical workforce development must accompany more immediate measures addressing the current surgical burden.
- The Lancet Commission on Global Surgery provides a framework to which children’s surgery can harmoniously be integrated.
- Innovative funding mechanisms may invest to scale cost-effective operations along with ongoing data collection and research.

## INTRODUCTION

The United Nations’ third Sustainable Development Goal (SDG-3) is to “ensure healthy lives and promote well-being for all at all ages.”^1^ In particular, this goal aspires to reduce neonatal mortality to less than 12 per 1,000 live births and under-5 mortality to less than 25 per 1,000.^2^ SDG-3 also addresses trauma, aspiring to halve “the number of global deaths and injuries from road traffic accidents” by 2020. Access to safe surgery and anesthesia will help achieve SDG-3 and will require focusing on low- and middle-income countries (LMICs),^3^ where more than 90% of child deaths occur.^4^ An estimated 43% of the population of sub-Saharan Africa is aged 15 and younger, and approximately 30% of the population in LMICs fall in this age group.^5^

Addressing the needs of this underserved community requires a coordinated “all hands on deck” approach between all stakeholders, particularly children’s health care providers, surgeons, and nonphysician clinicians. Global efforts addressing children’s health have historically, and to this day, focused their efforts on nonsurgical diseases (Figure).^6^^,^^7^

**FIGURE.**
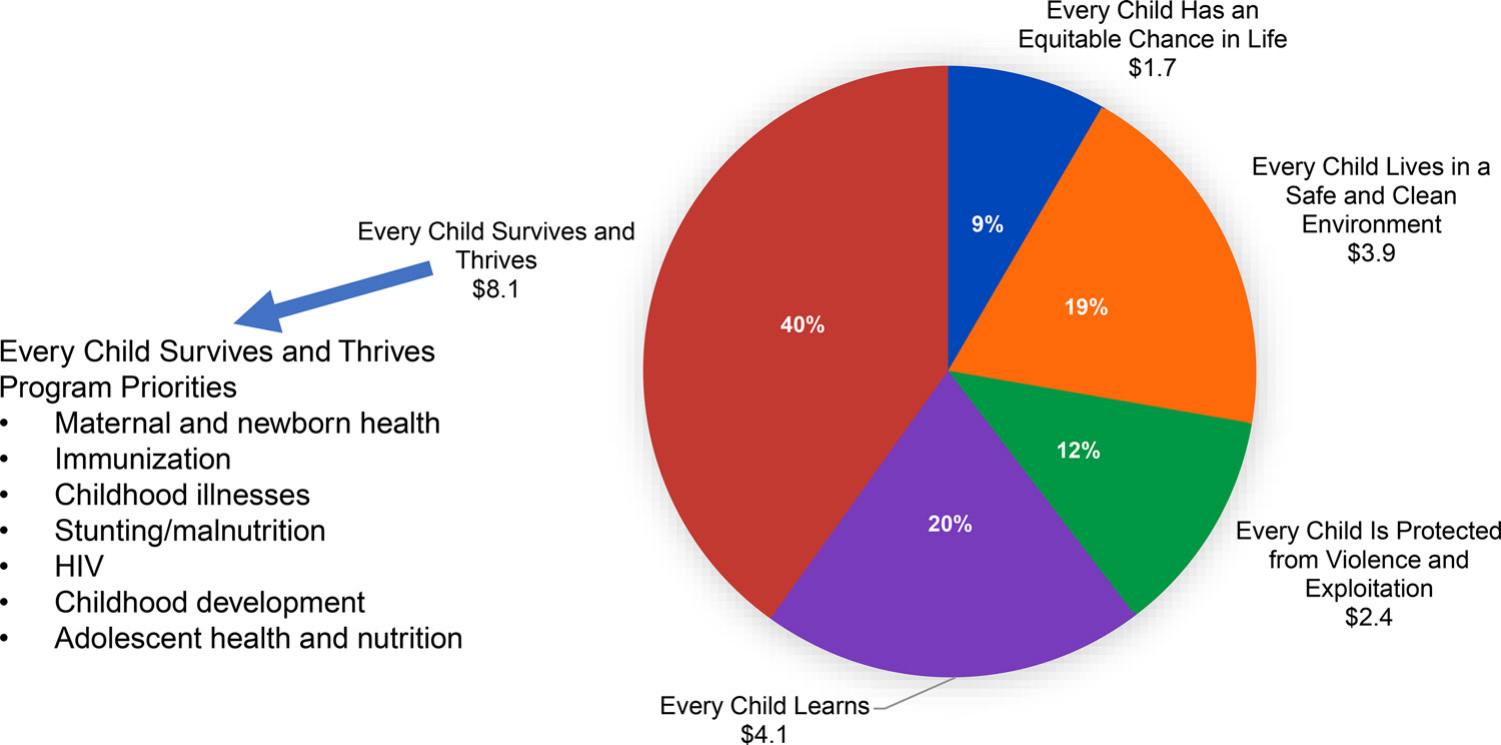
United Nations Children’s Fund Budget for Program Funding, 2018–2021^a^ ^a^ US$billion

## BURDEN OF CHILDHOOD SURGICAL DISEASE

Children and adolescents comprise 1.7 billion of the nearly 5 billion people who lack access to surgical care.^8^^–^^10^ To make matters worse, in some LMICs like The Gambia, estimates predict 85% of children will require surgical care before they are aged 15 years.^11^ Children’s surgery plays a large role in reducing the morbidity associated with noncommunicable conditions, such as inguinal hernias,^12^^,^^13^ injuries, and congenital anomalies,^14^ as well as various infectious disease complications, such as hydrocephalus and blindness from trachoma.^15^

Specific, surgical interventions for children have been found to be even more cost-effective than accepted adult surgeries, even in low-resource settings.^16^^–^^18^ In particular, the cost-effectiveness of circumcision has been reported to be similar to bed nets for malaria prevention.^17^ Additionally, the cost-effectiveness of cleft lip or palate repair, general surgery, hydrocephalus surgery, and ophthalmic surgery were all similar to that of the BCG vaccine for tuberculosis. Finally, the cost-effectiveness of cesarean deliveries and orthopedic surgery were more favorable than the cost- effectiveness of medical treatment for ischemic heart disease and HIV treatment.

Road traffic injuries alone, for example, account for more deaths in children aged 5 to 14 years than HIV, tuberculosis, and malaria combined.^19^ However, this burden is not borne uniformly across the world; 95% of all children killed by road traffic accidents are in LMICs.^20^ Similarly, 80% of all children killed by fall-related injuries are also in LMICs. Integrating children’s surgical providers into all levels of coordination—from supply chains to public health policies—is integral to addressing pediatric traumatic morbidity and mortality.

The gap between trained children’s surgical providers and the need is vast.

Cardiac, neural tube, and craniofacial anomalies, such as cleft lip and palate, account for at least 32 million lost disability adjusted life-years (DALYs), 57% of which could be averted through childhood surgical interventions.^15^ Currently, more than 300,000 newborns die within 4 weeks due to congenital anomalies.^21^ Studies examining the unmet burden of children’s surgical disease are lacking, but recent attempts to quantify and clarify these numbers have relied on using surgical delay and the resulting backlog of cases.^22^ In a study examining burden of congenital anomalies in 13 African countries, the average surgical delay was more than 2 years—contributing to nearly 75,000 lost DALYs.^23^^–^^25^ Surgical burden for congenital anomalies is likely related to not only surgical workforce, but also population size and birth rate—highlighting the need for collaboration between surgical and obstetric providers with public health practitioners.

Furthermore, this burden is spread among all LMICs, with sub-Saharan Africa and South Asia sharing a similarly large burden of DALYs avertable through children’s surgery.^26^ Clearly, more attention must be given to preventing, identifying, and treating congenital and noncommunicable diseases, injuries, and burns if the health of children is to be improved in accordance with global commitments.

## INADEQUATE SURGICAL WORKFORCE

The gap between trained children’s surgical providers and the need is vast. Currently, the number of children’s surgeons is *inversely* proportional to a country’s birth rate,^27^ meaning the countries that are most in need of surgical care for children have the least capacity for delivering this care. This need is most acute in many African nations, where the density of children’s surgeons ranges from 0.17 children’s surgeons per *million* children in Malawi, to 1.5 children’s surgeons per million in Egypt.^28^ Compared to a benchmark of 10 children’s surgeons per million children used by Krishnaswami et al., low-income African nations have a shortfall of more than 3,000 children’s surgeons.^27^ Equally essential, pediatric anesthesia faces severe workforce shortages, with specialized provider density estimated to be 100 times lower in LMICs than in high-income countries.^29^ To address this shortfall, LMICs need not only more anesthesia providers, but also providers who have specialty training and skills needed to manage pediatric anatomy and physiology.

## OVERLAPPING GOALS: PEDIATRIC AND GLOBAL SURGERY COMMUNITIES

The solidarity between children’s health care providers and children’s surgery providers is deeper than merely sharing patient populations and an overarching goal of improving children’s health. Children’s surgery would be ineffective without the children’s health care provider correctly diagnosing and referring a child with, for example, a congenital anomaly. Moreover, the presurgical preparation and postsurgical care for these patients, especially neonates, ideally would involve joint coordination between children’s health care providers and their surgical colleagues. Finally, synergies between humanitarian and health development exist—preparation for surgical care is an integral part to disaster and emergency preparedness around the world.^30^

Some progress toward addressing bringing children’s health care providers and surgery providers closer together has been made. In 2002, the Surgical Advisory Panel of the American Academy of Pediatrics worked with children’s health care providers to develop referral guidelines, representing an U.S.-focused example of potential collaboration.^31^ The Global Initiative for Children’s Surgery (GICS), founded in 2016, provides a platform and an organized voice for children’s surgery. GICS members—representing surgeons, anesthetists, and nonphysician clinicians—work with stakeholders in LMICs to identify barriers to care and develop country-specific plans to improve children’s surgical care.^32^^,^^33^ In addition, in 2013, the American Board of Pediatrics convened a Global Health Task Force to coordinate the expansion of their “core mission—training assessment, certification, and quality improvement and continuing professional development”—into the international sphere, helping to train international children’s health care providers through their International In-Training Examination.^4^^,^^34^ Although not specific to children’s surgery, the Global Health Workforce Alliance (now Network) focused on bringing attention to human resources for health to augment health care capacity.^35^

There is much to be gained from further integrating children’s surgery into advocacy efforts and the broader global child health agenda. Building off the history of collaboration between children’s surgeons and the American Academy of Pediatrics as early as 1948, the time has come to extend that relationship to the global sphere.^36^ In short, promoting good global child health requires both children’s health care providers and surgery providers to work together and outside of their specialty-specific silos.

Promoting good global child health requires both children’s health care providers and surgery providers to work together and outside their specialty-specific silos.

Much can be gained from integrating children’s surgery into advocacy efforts and the broader global child health agenda.

## RECOMMENDATIONS FOR IMPROVING GLOBAL CHILD HEALTH

### Develop the Children’s Surgical Workforce

An adequate surgical workforce is indispensable in meeting the demand for children’s surgical care. As both GICS and the Global Health Task Force have articulated, training a robust global pediatric workforce, with a focus on sustainable, ground-up improvements, is critical. One such model builds on the example set by the U.S. National Institutes of Health-funded Medical Education Partnership Initiative.^37^ Between 2010 and 2015, 13 medical schools in 12 sub-Saharan African countries were awarded $130 million to work with a U.S.-based university to increase the schools’ abilities to (1) produce more and better-trained doctors, (2) strengthen relevant research, and (3) retain graduates. Building on this model, children’s health care providers and children’s surgeon groups can collaborate with local medical schools and governments to support the development of a workforce specific for children’s health care in each country that integrates the provision of both medical and surgical care.

A survey of children’s surgeons in Africa demonstrated a clear preference for “collaborative professional development” over mission-based direct clinical care.^38^ The coordination of both pediatric- and surgery-specific organizations to improve in-country training opportunities and promote effective recognition and referral pathways for care is needed. These organizations should work with respective ministries of health, academic partners, and other stakeholders to develop local, pediatric-specific postgraduate residency training programs.

There exists a potential trade-off between the timeline necessary to sustainably develop and train a specialized workforce and the immediate clinical need today. Working closely with ministries of health, professional organizations, and existing referral networks, children’s health care providers and surgery providers should task shift and task share carefully selected components of care to nonspecialty trained providers as a potential, short-term bridge to the development of a robust and sustainable surgical workforce.^39^ Leveraging the critical importance of nonphysician clinicians is essential in achieving this “all hands-on deck” approach.^40^ Although large variations exist between countries, the volume of surgery performed by nonphysician providers can be significant: nearly 90% of obstetric surgeries and 39% of general surgery procedures—including 43% of nonobstetric laparotomies.^39^^,^^41^ Recent studies seek to assess the safety and efficacy of these surgeries.^42^^–^^44^ Future work must continue to focus on evaluating the outcomes of task sharing, as well as articulating best practices for supporting trained nonspecialty providers. Additionally, attention to retention of workforce is critical. Previous studies have suggested that surgical graduates in LMICs primarily migrate for “professional reasons.”^45^ Attempts through National Surgical Obstetric and Anesthesia Plans (NSOAPs, discussed later) to improve training alongside infrastructure may mitigate this problem.

Although secondary benefits of integrating efforts to increase a system’s surgical capacity for children exist to strengthen the health system as a whole, this approach requires sustained investment over time. In the more immediate term, there may be an ongoing role for selective, vertical programs to avert DALYs today, while broader health systems strengthening and capacity building are ongoing.^46^ In addition to the clinical impact of these surgical “camps,” potentials to engage with local infrastructure and workforce exist.^47^ Each country must decide whether to use this intervention, and every effort must be taken that these interventions are well regulated and do not detract from the longer-term goal of an increase in children’s surgical providers.

### Focus on “Best Buy” Surgeries

Children’s surgical care is cost-effective. In particular, inguinal hernia repair, trichiasis surgery, cleft lip and palate repair, male circumcision, congenital heart surgery, and orthopedic procedures are considered the 6 essential children’s surgical procedures because of the economic value for the health burdens they avert.^48^ These procedures, with the exception of congenital heart surgery, align with the 44 procedures deemed “essential” by the Disease Control Priorities Network, a joint enterprise devoted to determine disease control priorities around the world, particularly in LMICs.^49^

Although not traditionally considered as “children’s surgery,” reemphasizing the importance of cesarean deliveries must be included to address neonatal mortality and complications of pregnancy, the leading cause of death for girls aged 15 to 19 years.^50^^,^^51^ There is an inverse association between prevalence of cesarean deliveries and maternal and neonatal mortality for cesarean delivery rates up to 19%.^52^^,^^53^ The Disease Control Priorities Network noted that, as late as 2010, of the worldwide 16 million DALYs lost due to maternal disorders, 6.4 million DALYs were attributable to surgically preventable obstetric complications, including unsafe abortion.^54^^,^^55^

Children’s health care providers and surgery providers, alike, must work with obstetric providers to improve access to obstetric care and coordinate the appropriate referrals to optimize maternal and neonatal care. Through a focus on these cost-effective, “best buy” surgeries, the integration of surgical care can be sustainable, allowing for meaningful and lasting progress toward achieving the SDGs.

By focusing on “best buy” surgeries, the integration of surgical care can be sustainable and allow for meaningful progress toward achieving the SDGs.

### Integrate Children’s Surgery Into NSOAPs

In 2015, the Lancet Commission on Global Surgery proposed a framework for the creation of NSOAPs, providing an opportunity for governments to strengthen surgical care. Children’s surgery fits into this framework, and health officials should be encouraged to integrate children’s surgical care into both NSOAPs and national child health strategies. Nigeria has successfully included children’s surgery and nursing as a key component of their NSOAP.^56^ Additionally, children’s health care providers and children’s surgeons must collaborate to ensure that academic, governmental, and nongovernmental organizations working on various child health priorities communicate and collaborate, not only with one another, but also with ministries of health. Moreover, enabling the environment for children’s surgery requires attention to diverse domains, including infrastructure, blood supply, infection control, and quality improvement. The NSOAP provides a mechanism for achieving this whole-of-systems approach.^57^ Using the Lancet Commission’s framework, all pediatric-focused groups must advocate for the inclusion of pediatric-specific interventions at the government level into national and regional health planning using the GICS Optimal Resources guide.^32^

### Standardize Data Collection and Research

Planning and effectively incorporating children’s surgery into national health systems is not possible without adequate and reliable information. Recent strides have been made to improve data collection around children’s surgery,^58^^–^^61^ but these efforts must be scaled, standardized, and aligned with existing surgical indicators as described by the Lancet Commission on Global Surgery (i.e., children’s surgical volume, access to care within 2 hours, workforce density, financial risk protection, and perioperative mortality).^62^ Building off existing initiatives such as the Quality of Care Network to encompass more children’s health areas is a potential way to scale and standardize these efforts.^63^ Additionally, baseline assessments of the surgical services available at hospitals, such as through the District Health Information Software or Service Provision/Service Availability and Readiness Assessments,^64^^,^^65^ need to be updated and emphasized to specifically include children’s surgery.^66^ A combined effort within the global child health community can help standardize data collections, distribute analyses, and collaborate with in-country providers to set country-driven research agendas. Developing a nation’s capacity to gather and analyze their own children’s health data will improve all areas of children’s health care.^67^

### Leverage Financing

Harnessing the ethical, health, and economic arguments for investing in children’s health, which necessarily includes surgery, requires public, private, and academic partnerships. Given their impact on development, untreated pediatric and surgical conditions carry an additional burden of lifelong disability and ensuing economic disadvantage. Surgery is a cost-effective global health intervention; thus it is essential that both public and private funders incorporate surgical care within the package of services aimed at promoting global child health.^17^ One particular avenue is through the World Bank’s Global Financing Facility for Women, Children, and Adolescents, whose ultimate aim, through investment cases in maternal and child health, is to “gradually shift countries away from relying on developmental aid and onto a sustainable financing path.”^68^ Helping to champion pediatric surgical priorities among existing funders of children’s health is an area where children’s health care providers, with more experience in navigating funders and funding mechanisms, can drive global access to children’s surgery forward.

## CONCLUSION

Addressing preventable neonatal and child deaths and achieving SDG-3 requires a coordinated approach between children’s health care providers and the surgical and anesthesia providers. Global child health demands surgery, and timely surgery, in turn, arrives only with the support and collaboration between children’s health care providers and their colleagues. By aligning advocacy and fundraising efforts to include specific, cost-effective, and necessary surgeries, the global child health care and surgery communities can more effectively partner with countries to achieve this goal and offer comprehensive children’s health care to those who need it most.

## Notes

**Peer Reviewed**
